# 

**Published:** 1891-01-03

**Authors:** 


PRICE ONE PENNY.

No. 223, Vol. IX.—January 3rd, 1891.

[Registered at the General Post Office as a Newspaper for transmission in the United Kingdom and Abroad.]

WITH EXTRA NURSING SUPPLEMENT

Per Annum, 6s. 6d.; post free.

Monthly Parts, 6d.; post free 8d.

Ditto per Annum, 8s., post free.

### THE HOSPITAL

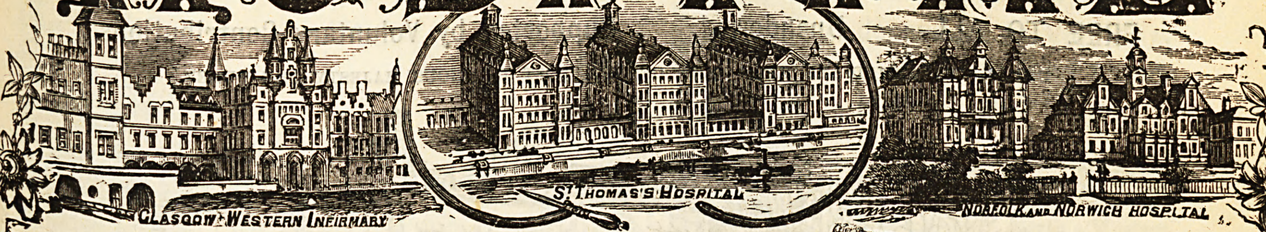

A WEEKLY JOURNAL OF

Science, Medicine, Nursing and Philanthropy.

### NEW YEAR'S APPEAL NUMBER.

SATURDAY, JANUARY 3rd, 1891.

**Who Are the Philanthropists?** - 213
**Passing Topics.**—The C. O. S. as Collector for Charities - 214
**1890: A Various Year.**—The Registration of Midwives—Nurses' Registration—The National Pension Fund—Hypnotism, 215; Pasteur and Hydrophobia—Mr. Gladstone: Sir George Stokes—Black Clouds over Hospitals—The Dead—Koch and the Consumption Cure, 216; Epilogue - 217
**The Luxury of Giving** - 217
**Annotations.**—The Living Workmen and the Dead Archbishop—Boy and Girl Marriages—The Vice of Pity - 218
**The Philanthropist's Vade Mecum.**—General Hospitals, 217; Hospitals for Consumption, 218; Hospitals for Children—Pay Hospitals, 219; Lying-in Hospitals—Nervous Diseases, Epilepsy, and Paralysis, 220; Miscellaneous Special Hospitals—Nursing Institutions, 221; Asylum for Idiots—A Few Other Charities - 222

### EXTRA SUPPLEMENT—THE NURSING MIRROR.

**En Passant.**—St. Helena Home—Short Items—Looking Forward—Sweet Innocence—New York Hospital—Eighteen-Ninety - lxxi

**Lectures on Surgical Ward Work and Nursing**—By Alex Miles, M.B.Edin., C.M., F.R.C.S.E.—Lecture XVIII.—Wounds and Cleanliness - lxxii

**Nursing Medals and Certificates.**—No. III.—The Mary Adelaide Medal - lxxiii

**For Reading to the Sick.**—Snow - lxxiii

**Distributing Christmas Parcels** - lxxiv

**National Pension Fund for Nurses.**—A New Year's Gift - lxxiv

**Presentations** - lxxiv

**Everybody's Opinion.**—Diphtheria and the Use of Brandy—Scotch Infirmaries—Fever Hospitals—The Trained Nurse in the Nursery - lxxv

**Appointments—Notes and Queries** - lxxv

**Off Duty.**—The Brimsby Ghost - lxxvi

**Where to Go.** - lxxvi

**Amusements and Relaxation** - lxxvi

